# Letters and Answers to Correspondents

**Published:** 1874-01

**Authors:** 


### Our Correspondence.

### Letters Exposing Humbugs and Swindlers,

With other Communications of Interest to the People.

EASTON, PA., NOV. 20, 1873.

DEAR DOCTOR:

What is good for a scalp that itches? C.

Scratch it!

---

TROY, N. Y., NOV. 8, 1873.

DR. UP DE GRAFF:

I have a little boy, six years of age, who has what physicians call a spinal curvature. He has been in New York under the care of Dr. ———, for the past six months, who has given him tonic treatment, rubbed him with stimulating liniments, and kept him in bed most of the time. I cannot see that he is a particle better, in fact I believe he grows worse. I saw a little child recently that has been under your care with the same complaint and am so induced to write to you. Do you think you can cure him?

L. A. B.

Cannot tell without first seeing your child. Read the October number of the BISTORY for our opinion of and mode of treatment of spinal curvatures.

---

CANANDAIGUA, N. Y., NOV. 18, '73.

EDITOR BISTOURY:

I have seen the Bistoury frequently at the office of our family physician, have seen your answers to correspondents, and therefore write you. I am troubled with nervous debility, have read a description of my symptoms in a pamphlet published in New York by Drs. Jourdon & Beck. Believe they call it *spermatorrhœa.* I have sent them money for medicine, also Dr. J. B. Ogden, Rev. Henry A. Wilson. I have taken gallons of medicine, for which I have paid more than a hundred dollars, and am not in the least benefited. I don't mean to give any other doctor a chance to swindle me out of my money without he can cure me. Now, what I want to know is, can you do me any good. If so I am willing to pay for it. Address,

———————

Can we do you good? Of course we can, and through this advice. Cease writing to or spending money upon such men as you have named. They are wretched frauds, who reap a rich harvest by inveigling into their clutches just such green-horns as you. Whenever you are promised through an advertisement, medicines or advice free, set the advertiser down as a swindler. Can't you see that no man can afford, nor are there any benevolent enough, to offer you something for *nothing*, and pay for advertising such a proposition, into the bargain? You must be a great noodle not to know better than that. Of course, their medicines did not benefit you. That is not surprising! They are composed only of salt and colored water. Never knew salt to cure anything but dead meat, and you say *you* are alive, although you don't act much like a remarkably animated chap.

Your determination not to be "swindled out of your money *without* being cured," is a wholesome one; stick to it.

---

TUSCUMBIA, ALA. Dec., 1, 1873.

DR. UP DE GRAFF:

I saw the Bistoury for the first time yesterday. I like it much, for the manner in which you deal with medical rogues. I enclose one dollar as subscription for myself and Dr.——. Please place us on your list, We have a traveling mountebank in this region, who goes from village to village announcing himself as "Old Doctor Blink." He deals in "roots and yarbs," gives no "quicksilver" or other "allepathy stuff," and is great on tape worms. He can get a tape worm, at least 60 ft. long, from any one who may have the courage to take his remedy (?) Strange to say he finds plenty who are willing to patronize him, and I was called to a man yesterday who had actually eaten a pint,—an entire pint, of cowage mixed to the consistence of a paste, with castor oil. This nauseous dose the poor fellow swallowed, how, the Lord only knows, and followed it with several doses of castor oil to carry it off. When I saw him he was so faint and exhausted from the consequent cathartism as to be almost pulseless, while his wife pointed to a vessel which she said contained the tape worm. We examined it and found it to be shreds of mucus membrane, literally torn from stomach and intestines by the cathartic. We learn that this is the method he adopts with all his patrons, and of course, always is able to produce a worm of the character seen in the case mentioned. Which is needed most, a law for the suppression of quackery, or the punishment of willful ignorance?

J. P. L., M. D.

Human frailty is the same everywhere. In no special department is it more exhibited than in the selection of means of cure for some real or imaginary disease. Whether it be in the acceptance of the Allopathic formulæ, the homeopathic globule, or the sulphurous recipe from the mysterious realms of the damned,—all have their patrons and believers,—all declare themselves cured. After all, it matters but little *how* a man is cured provided he believes his selection of means afforded the sought for relief. When he selects a pint of cowage and castor oil, and swallows it, as an indication of his faith, he *deserves* to get well, even if he made a contemptible ass of himself in his endeavor.—We believe in allowing people to act pretty much as they choose, in selecting their means of medication. The more they are opposed or ridiculed, the more determined they seem to be in converting their stomach and bowels into a sort of experimental laboratory, for the benefit of all classes of quacks and impostors.

---

Covington, Ky., Oct 20, 1873.

Dr. Up de Graff :

I sent you a man, Mr.——, a month ago, to be operated upon for *cataract*. Did he reach you, and what has become of him ?

J. R. T., M. D.

Operated upon, well, and gone home.

---

Horseheads, N. Y., Dec. 12, 1873.

Dr. Up de Graff.

Do you know anything of Dr.——? He came to our house yesterday and said he could cure a fever-sore on my leg. I have learned that he makes inquiry from house to house for persons suffering with any chronic disease. He offered to cure me for $5. Do you know anything about him, and would you trust him ?

M——.

By all means ! Give him your five dollars quickly, and so exemplify the adage, "the fool and his money, etc." He must be a very celebrated physician. Doubtless has written several works on heathen mythology, (*you* should read them,) and is, perhaps, a member of the Anglo-Saxon Fever-sore Society. Distinguished ! of course he is ! That is precisely the manner in which learned physicians secure their patrons. They search for them from door to door. Why, yes, set him to work at once, and when he gets your leg ready to saw off, come down and see us, we will afford you our services cheap, very—out of consideration for your verdancy.

---

Artificial Butter.—It has long been known that butter is manufactured for the London market from any refuse grease that can be obtained, but it is only recently that the manufacture of butter from tallow has been attempted in this country. The process is a very simple one, and consists in making an emulsion of tallow oil by aid of sour milk, and coloring it with a little annatto. A large company has been organized for the purpose of making this butter for the New York market, and we hear rumors of another nearer home, the building for the purpose having already been erected in Cambridge.

The operation is as follows. Fresh beef suet is hashed fine and then rendered in open tanks, by means of steam heat. The clear fat is drawn off from the top of the tank and allowed to cool in large vessels. By slow cooling the fat crystallizes, and the more solid margarine and stearine separate from the oleine which remains diffused through the mass. The semi-solid mass is then put into cotton bags, as in the ordinary process for the manufacture of lard and tallow oil, the only exception being that rather more attention is paid to cleanliness. These bags, which hold about two pounds each, are then placed in a powerful press which expresses the oil, As it flows from the press, the oil is clear, yellow in color, tasteless, and without odor, if sufficient care has been used in its preparation, The residue in the bags is used for making candles, Thus far there is nothing new in the process,

The oil is placed in churns with one fifth of its weight of sour milk, and churned until an emulsion is formed, sufficient annatto having been added to give it the required color, It is then cooled down and worked and salted like ordinary butter. By churning it takes up nearly one third of its weight of water. It is claimed that this butter will keep in any climate, and that it cannot be distinguised from good firkin butter. It is said that some of the hotels in New York are using the article. The manufacture was introduced into this country by M. Paraf, and the New York company are turning out over two tons per day. The farmers need not be at all alarmed, for good dairy butter still brings 38 to 40 cents per pound in the Boston market and there seems no likelihood of its being lower. *Boston Journal Chemistry.*

